# Knowledge, attitudes and practices toward breast cancer screening in a rural South African community

**DOI:** 10.4102/curationis.v38i1.1172

**Published:** 2015-02-27

**Authors:** Dorah U. Ramathuba, Confidence T. Ratshirumbi, Tshilidzi M. Mashamba

**Affiliations:** 1 Department of Advanced Nursing, University of Venda, South Africa; 2 Department of Psychology, University of Venda, South Africa

## Abstract

**Objectives:**

The study assessed the knowledge, attitudes and breast cancer screening practices amongst women aged 30–65 years residing in a rural South African community.

**Method:**

A quantitative, descriptive cross-sectional design was used and a systematic sampling technique was employed to select 150 participants. The questionnaire was pretested for validity and consistency. Ethical considerations were adhered to in protecting the rights of participants. Thereafter, data were collected and analysed descriptively using the Predictive Analytics Software program.

**Results:**

Findings revealed that the level of knowledge about breast cancer of women in Makwarani Community was relatively low. The attitude toward breast cancer was negative whereas the majority of women had never performed breast cancer diagnostic methods.

**Conclusion:**

Health education on breast cancer screening practices is lacking and the knowledge deficit can contribute negatively to early detection of breast cancer and compound late detection. Based on the findings, community-based intervention was recommended in order to bridge the knowledge gap.

## Introduction

Breast cancer is a global health problem and the most common cancer amongst women, comprising 23% of the female cancers (Parkins & Fernández 2006:S71). It is also the leading cause of cancer-related deaths in low-resourced countries. Women in any age range are at risk of breast cancer and the risks increases with advanced age (Omotara *et al*. 2012:1). Despite the development of advanced technology in the detection of breast cancer, the mortality rate remains high. Breast cancer is the main cause of cancer mortality in women aged 40–44 years old (Alwan *et al*. 2012:338; Khanjani, Noor & Rostami 2012:177). Although substantial improvement in survival has been reported in high-income countries, the risk continues to increase. The survival rates in middle- and low-income countries remain low (Alwan *et al*. 2012:338).

Data from South Africa's National Cancer Registry (NCR) show breast cancer as the leading cancer amongst women. South African women have a one in 29 lifetime risk of developing breast cancer, with an age-standardised incidence rate of 30.6 per 100 000 population (Lemlem *et al*. 2013:2). In a study done in Turkey amongst female health workers, it was found that breast self-examination (BSE) was not a regular behaviour; doctors made up 31.3% of those who performed BSE and midwives, 21.8% (Akpinar *et al*. 2011:3065). This indicates that health personnel do perform BSE, but the rate of those who practise BSE on a regular basis is low. The level of knowledge and attitude of health professionals are important determinants for the use of screening programmes and creation of an environment supportive of screening behaviours by offering positive role models. Empowering nurses with information about early detection methods and their related benefits could help advance their skills in performing BSE and expand their roles as client educators (Akpinar *et al*. 2011:3065; Lemlem 2013:3). It is important that health personnel are aware of the risk factors for breast cancer, in order to guide their patients for necessary screening.

Lack of basic knowledge and an effective information delivery system for breast cancer further threatens the life and well-being of women. Breast cancer is silently killing women – mainly those who have no knowledge and continue to be ignorant about breast cancer and breast diagnostic screening methods for early detection (Shepherd & McInerney 2006:71). Knowledge is a necessary component but it is insufficient unless the cultural relevance is assured by the health professional providing direct healthcare (Dow Meneses & Yarbro 2007:111). Omotara *et al*. (2012:1) also reported lack of information regarding breast cancer to the rural and urban populace of Nigeria, saying that it is responsible for the negative perception of the curability of a cancer detected early and the efficacy of screening tests. In addition, silence and lack of understanding of the concept of risk factors associated with breast cancer discourage people from seeking early intervention or even admitting that the symptoms that they are experiencing are related to breast cancer. Level of awareness regarding how to perform simple life-saving diagnostic breast checks such as BSE further compounds the problem of late detection. Empowerment of women with information on BSE is of paramount importance, especially in countries without modern technologies for breast cancer screening (Shepherd & McInerney 2007:38). South African rural communities have limited technological resources, but BSE can contribute greatly if women are informed about this technique and regular practice would reduce late presentation.

Akhigbe and Akhigbe (2012:74) also suggest that health beliefs differs from culture to culture, that cancer fatalism may be a deterrent to participation in health-promoting behaviours. This is because some people believe that illnesses or catastrophic events happen because of a higher power (such as God), or they are meant to happen and cannot be avoided; as a result, fatalism becomes part of the person's worldview. Black African communities usually associate chronic conditions with witchcraft and evil spirits. Cultural values and ethnic diversity have an impact on health beliefs, which may influence how rural women interact with the western medication, especially conditions such as breast cancer. Some women delay seeking treatment because of fear of stigma concerning their daughters as it is believed that they also might be affected by breast cancer and might not be considered for a good marriage. Furthermore, it is believed that cancer is a death sentence from God (Dow Meneses & Yarbro 2007:108).

### Problem statement

Breast cancer is the most common cancer in South Africa and it is increasing in incidence, with large numbers of women with breast cancer being found in rural areas. The reason for this could be that access to information is limited as many women miss out on early detection because of their lack of knowledge and practice of BSE and other screening practices (Khokhar 2009:249). The researcher observed a pattern of women presenting with symptoms of late stage of the disease, such as a red, swollen, tender breast, but confusing it with an inflammatory condition. This is despite the fact that breast cancer is a preventable disease, which indicates a knowledge deficit. When a woman notices skin changes on the breast, she should immediately seek medical help for early diagnosis and treatment. In Thulamela Municipality, for the year spanning 2011–2012, there were 85 women being treated for gynaecological cancers at the Provincial hospital, according to the gynaecological register in two hospitals within the district for the financial year 2011 and 2012 (District Health Statistics-2011/2012). There are different methods for early diagnosis of breast cancer: BSE, physical examination by a medical doctor and mammography. Unfortunately, most breast cancers are diagnosed when they are in the advanced stages. Breast cancer mortality in Vhembe District may be compounded by limited resources, inadequate preventative screening programmes and lack of access to advanced technology in rural health facilities, leading to late presentation or not coming forth because of their health-belief system.

#### Purpose

The purpose of the study was to determine the knowledge, attitudes and breast cancer screening practices amongst women in a rural South Africa community.

#### Objectives

To assess knowledge regarding breast cancer amongst women in a rural South African community.

To describe the attitudes and practices regarding breast cancer screening amongst women in a rural South African community.

## Research methods and design

### Research setting

Vhembe District is one of the districts in Limpopo Province, situated in the northern part of the province and sharing borders with the Capricorn and Mopani districts in the eastern and western parts, respectively. The sharing of borders extends to Zimbabwe and Botswana in the northwest and Mozambique in the southeast, through the Kruger National Park. Vhembe District comprises three municipalities, namely, Thulamela, Makhado and Mutale. The study was undertaken in Makwarani, a rural village in the outskirts of Thohoyandou town, which is a low-resourced Venda community in Thulamela Municipality.

### Research design

This study employed a descriptive cross-sectional survey, which is a non-experimental design examining data from a specific group, at one point in time (LoBiondo-Wood & Haber 2006:244), designed to assess knowledge, attitudes and practices toward breast cancer screening in a rural South African community.

### Population

Burns and Grove (2007:40) state that a population comprises all elements (individuals, objects or substances) that meet the criteria for inclusion in a study. In this survey, the study population comprised 150 women. The target population was all Tshivenda-speaking women aged between 30–65 years.

### Sampling

Systematic sampling was used for this study. Polit and Beck (2008:347) state that the process of systematic sampling involves selecting every *k*th individual on the list after having selected a starting point at random. Makwarani village has 300 households; these were written on a list and 150 households were selected randomly using the lottery method, with a random start of two and then the researcher selected one woman from every second household, which comprised 150 women. This type of sampling was used because probability sampling reduces sampling errors and bias, whilst enhancing representation and the confidence of the sample.

### Data collection method

The researcher used a closed-ended questionnaire comprising three sections, namely, demographic information, knowledge about breast cancer and breast cancer screening practices. A Likert scale was used to gather information on attitudes toward breast cancer. The questionnaire was translated into the local African language (Venda) for easier comprehension by the participants. This was done by Venda educator in the Department of African Languages at the University. There was a back translation by another language educator in order to ensure English and Tshivenda equivalence. One hundred and fifty questionnaires were self-administered and were collected after three days from the respective households, with a response rate of 100%.

### Data analysis

Statistical analysis was used to summarise the results of the study and to reduce, organise and give meaning to the data (Burns & Grove 2005:43) obtained from the 150 completed questionnaires. A statistician analysed the data by using the Predictive Analytics Software (PASW) version 18.0 (SPSS Inc., Chicago 2009) and Microsoft^®^ Excel was used to draw some charts. Descriptive statistics were used to describe the data and the Chi-square test was used to describe relationships amongst variables, with a significance level of *p* < 0.00.

## Ethical considerations

Ethical clearance was obtained from the University of Venda Ethics Committee (project SHS/11/PH/004). Authorisation and approval for conducting the study were requested from Makwarani Royal Committee. The researchers ensured protection of the rights of the participants by informing the participants that their participation was voluntary, they would remain anonymous, they were free to withdraw from the study at any time without penalty and that all information would be kept confidential. Each participant signed a written consent; even those with no formal education were able to write down their names.

## Trustworthiness

### Validity and reliability

Content validity concerns the degree to which the instrument has an appropriate sample of items for the construct being measured (Polit & Beck 2008:458). The questionnaire was scrutinised for relevance by peers in the field of oncology nursing; construct validity was ensured by aligning the content of the instrument based on literature related to oncology and reproductive health. Reliability refers to the consistency and stability with which the instrument measures a target attribute, if administered to different individuals at different times (Polit & Beck 2008:455). Reliability of the research instrument evaluated by use of a pre-test. Fifteen questionnaires were distributed to women who were not part of the study in order to test the stability of the instrument, including clarity of the questions. The questionnaire addressed the respondents in their own language so as to improve understanding.

## Results

### Socio-demographic characteristics

The score for the participants’ mean age was 1.6, with a range of 30 years to 65 years. Forty (26.7%) of the participants had no formal education, whilst only 26 (17.3%) had a tertiary level of education, 47 (31.3%) had a secondary education and 37 (24.7%) had obtained a primary-level education. Twenty-six (17.3%) were professional workers whilst 50 (29.3%) were casual labourers and pensioners.

### Knowledge on breast cancer and source of information

One hundred and four (69%) of the women had never heard of breast cancer, whilst only 46 (31%) had heard of breast cancer, with their source of information being mainly from the media (*n* = 26; 56%) as opposed to coming from a health facility (*n* = 6; 14%).

### Symptoms of breast cancer

Swelling of the breast or lump was cited by the majority of participants (*n* = 90; 60%), followed by abscess (*n* = 25; 17%) and accumulation of fluid in the breast (*n* = 15; 10%). Nipple changes (*n* = 10; 7%), skin changes (*n* = 5; 3.3%) and swelling of the breast or armpit (*n* = 5; 3%) were least known.

### Risk factors for breast cancer

The responses to the list of risk factors for breast cancer were as follows: high fat intake (*n* = 32; 21.3%), lack of exercise (*n* = 30; 20.0%) and overweight (*n* = 22; 14.7%). Others, in order of percentage, were: hormonal changes (*n* = 19; 12.8%), heredity and radiation (both *n* = 14; 9.3%), drinking alcohol (*n* = 13; 8.7%) and smoking (*n* = 6; 4.0%).

### Knowledge of breast cancer diagnostic methods

Eight (5.3%) of the participants had heard about breast diagnostic methods, whereas the majority (*n* = 142; 95.0%) of the participants never heard about breast diagnostic methods. Amongst the eight (5.3%) participants who said they had heard about breast cancer diagnostic methods, four (2.6%) knew of clinical breast examination, three (2%) knew of BSE and one (0.6%) mammography. Furthermore, 94 (62.5%) of the participants did not know the appropriate time to perform BSE, whereas 56 (37.5%) knew that BSE should be performed seven days after menstruation.

### Attitudes toward treatment and early detection

Almost half of the women (*n* = 75; 50%) disagreed and 35 (23.3%) strongly disagreed that breast cancer treatment worsens the condition, whereas 33 (22.0%) agreed and 7 (4.7%) strongly agreed that the treatment worsens the condition. Seventy (46.7%) of the women agreed that the possibility of a cure for breast cancer is determined by early detection and 38 (25.3%) strongly agreed with this statement, whereas 30 (20.0%) disagreed and 12 (8%) strongly disagreed that the possibility of a cure is not determined by early detection.

### Practices of breast cancer screening and behaviour

One hundred and forty-two (94.7%) of the women said that they had never performed breast cancer diagnostic checks and only eight (6.3%) had practised one of the methods during their lifetime. Regarding health-seeking behaviour, 124 (82.7%) reported that if they noticed any change in their breast, they would consult the medical doctor, 21 (14.0%) said that they would consult the traditional doctor and only five (3.3%) would consult a prophet.

## Discussion

Table 1 provides the demographic profile for the study (*N* = 150) and describes the age distribution, level of education and occupational status. The items in this section attempted to obtain personal information about the participants in order to contextualise the responses concerning their knowledge, attitudes and practices regarding breast cancer.

**TABLE 1:** Demographic characteristics.

Characteristics	*n*	%
**Ages**
30–39	80	53.3
40–49	55	36.7
50–59	5	3.3
60–65	10	6.7
**Level of education**
No formal education	40	26.7
Primary education	37	24.7
Secondary education	47	31.3
Tertiary education	26	17.3
**Socioeconomic status**
Unemployed	80	53.3
Professional	26	17.3
Self-employed	20	13.3
Domestic worker	20	13.3
Pensioner	4	2.7

The strongest risk factor for breast cancer is age. A woman's risk of developing this disease increases as she gets older, however those who are at risk should be made aware of personal risk factors of developing breast cancer. Similarly, the participants in the older age group displayed low levels of knowledge regarding screening practices. Allam and Abd Elaziz (2012:196) concur with the findings that younger subjects in Egypt had a higher level of knowledge about breast cancer compared with older subjects. When older women demonstrate poor knowledge of breast cancer, it is of great concern as the risk of cancer increases with age. It is at this age that women should be more proactive in their health promotion behaviours. Women in the menopausal stage are at risk as a result of the high levels of hormones associated with breast cancer.

Employment status determines one's ability to access healthcare, as well as determining other factors in terms of cost, proximity and acceptance of medical services. Occupation and education show a positive association with breast cancer screening methods; women of intermediate and high occupational class are more likely to use screening methods as compared with those in the lowest class (Damiani *et al*. 2012:2). Socioeconomic status may determine the variety of life styles and dietary practices that might affect breast cancer risk, as well as possibly influencing the health-seeking behaviour of the participants. The majority of women in the study were of low socioeconomic status which is linked to decreased rates of breast cancer screening, greater probability for late-stage diagnosis, receipt of inadequate and disparate treatment and higher mortality from breast cancer. Poverty is associated with poorer breast cancer outcomes worldwide and only 26 (17.3%) of the participants were working professionally. The possibilities of these women going for screening is limited if the information is not available or services are limited. This might also increase their mortality rate as they might also present late for screening, resulting in poor health outcomes.

Education is also a marker for specific traits such as intelligence, acquisition of adaptive skills, or awareness of risky health behaviours; it improves our understanding of the causes and natural history of some diseases. The Chi-square test was carried out to determine the relationship between level of education and breast cancer screening practices. The mean score for the level of education was 1.4, with a *p*-value of < 0.00, which suggests that those who are educated were likely to use breast-screening methods, unlike the uneducated. Education might influence the participants’ knowledge of breast cancer as well as the methods of breast cancer early detection. Alwan *et al*. (2012:343) report that breast-screening practices appear to be correlated with the higher level of education and healthcare services offered in those regions as compared with the developing world. However, Reyes-Ortiz *et al*. (2007:392), surprisingly, reported that a few college women were aware of the procedures but did not practise them. Similar findings were reported by Khokhar (2009:248), where teachers in Nigeria were aware of breast-screening practices but none of them knew that clinical breast examination (CBE) and mammography should be done annually from the age of 40 years; BSE was not performed regularly by the participants in the study. In this study, women lacked adequate information on breast cancer, symptoms and breast-screening practices. Education plays a vital part in modifying lifestyle behaviour, however health education and promotion are important in reinforcing breast cancer screening practices through community awareness campaigns so as to empower women to take a proactive approach to their health.

Knowledge is equally important as it could influence health-seeking behaviour and change attitudes toward breast cancer. McMullin *et al*. (2008:33), in their study conducted in United States amongst a Togan population sample, reported that 35 of the 48 participants lacked knowledge of exactly what cancer is, although they knew that the disease was linked to death. Some compared it with HIV, saying it is a disease for which there is no cure. This understanding of cancer as a death sentence is often informed by cancer experiences on the part of family and friends. Another study conducted in South Nigeria reported that the mean knowledge score was about 42.3% and only 21.4% of the participants were aware of breast cancer (Omotara *et al*. 2012:2). This might be attributed to ineffective awareness campaigns by health workers, as they should be helping women to develop healthy lifestyle practices and promote breast-screening practices. The main source of information to the participants was the media (56%), whilst clinics and/or hospitals provided 14%. This suggests that hospitals and clinics are not doing enough to make information readily accessible to the communities and, as such, women rely on media such as radio and television which do not address health issues in a constant manner. The media coverage is sporadic and health promotion issues are only highlighted during awareness days or weeks, which is not effective. Thus, health education should be intensified so as to enlighten women of all age groups on the particular risks of breast cancer. Omatara *et al*. (2012:2) are also of the opinion that health workers should endeavour to educate women on breast awareness during regular clinic visits for other health issues in order to increase the level of awareness of breast cancer in the community. Most women either lack knowledge or are ignorant about breast cancer; they usually not perceive themselves as being susceptible or at risk of breast cancer, especially amongst black communities. They see cancers as being diseases that affect other racial groups.

**FIGURE 1:**
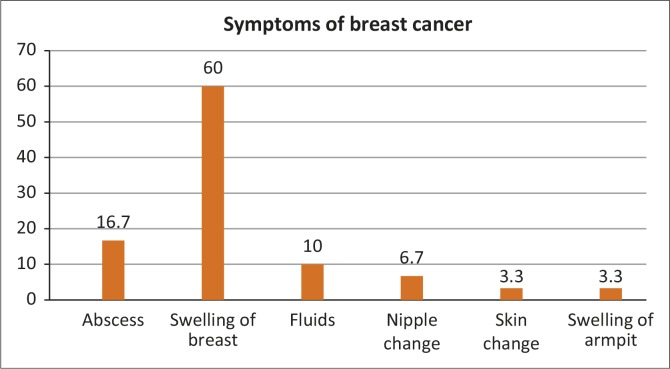
Responses regarding symptoms of breast cancer.

Black women historically have had fewer incidences of breast cancer as compared with white women and there are still those who are unaware that, as a result of lifestyle changes, they are at risk of developing breast cancer. They need to understand how to detect the disease early – when they feel the lump they should not ignore it. Figure 1 depicts the low levels of knowledge regarding symptoms, such as accumulation of fluid in the breast, nipple changes, skin changes and swelling of the breast or armpit. The findings of this study contrast with studies in Botswana and Brazil. Tieng'O *et al*. (2011:3524) and Marinho *et al*. (2008:25) reported that 54.1% and 42.4% of participants, respectively, could identify that nipple discharge and skin changes are symptoms of breast cancer. Insufficient knowledge about the signs and symptoms of breast cancer might lead to poor performances of breast diagnostic checks. Knowledge about the symptoms of breast cancer appears to influence the participants’ screening behaviour. It can thus be concluded that knowledge is a basic component that may help the participants to perform breast cancer diagnostic checks for early breast cancer detection. Poorer knowledge regarding the identification of symptoms of breast cancer is associated with late detection and poor survival.

**FIGURE 2:**
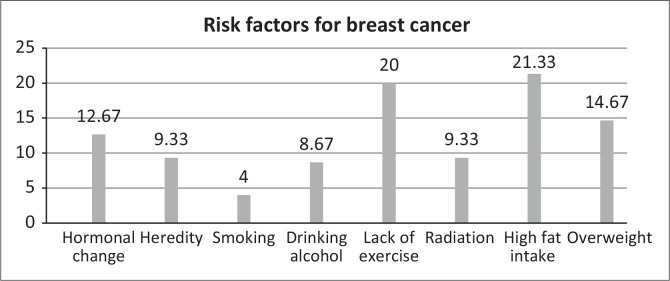
Risk factors for breast cancer.

Most women do not perceive themselves as being at risk for breast cancer as the information regarding risk factors is not readily available in rural areas. Figure 2 indicates that risk factors such as smoking, genetics, hormones and obesity were not known, although obesity is common amongst black women. The findings are consistent with those of Al-Dubai *et al*. (2011:2536) who also reported poor understanding of risk factors and unsatisfactory responses amongst Malaysian women, where nulliparity, delivery at older than 30 years, big breasts, contraceptive pills and menarche before age were not known to be risk factors. Yan (2009:100) also found that breastfeeding, age of menopause and menarche were not recognised as risk factors; these findings may lead to underestimation of the importance of regular screening by older women if they believe that it is the younger age group that is most at risk. The results of the survey suggest the need for educational programmes to improve current knowledge of cancer. Knowing the risk factors for breast cancer might help the participants to adopt a healthy lifestyle of proper nutrition and exercise, avoidance of unnecessary exposure to radiation and going for genetic testing if at risk, as well as avoiding such practices such as smoking and drinking in order to reduce the incidence of breast cancer morbidity and mortality (Hadi *et al*. 2010:33). Poor knowledge of risk factors and knowledge of their relative risk of developing breast cancer also explains why they do not engage in health-promotion behaviour or breast-screening practices.

Poor knowledge regarding breast cancer screening methods is responsible for late detection of the disease. Eight (5.3%) of the participants had heard about breast diagnostic methods, whereas the majority (*n* = 142; 95.0%) of the participants had never heard about breast diagnostic methods. Knowledge about diagnostic methods is highly beneficial to the participants as it assists women regarding when and how to perform breast cancer screening for early detection in order to reduce late presentation of the disease that might complicate treatment of breast cancer. Furthermore, 62.5% of the participants did not know the appropriate time to perform BSE, whereas 37.5% knew that BSE should be performed seven days after menstruation. Kanaga *et al*. (2011:1967) concur that the low rates of BSE, CBE and mammography were also of concern amongst Malaysian women and suggested that increased awareness and subsidised mammography be given to the general population. The Minister of Health in South Africa also intends to adopt the National Health Insurance so as to increase accessibility of such diagnostic screening tests. Lack of social support can influence the choice of screening amongst women.

One hundred and thirty-one (88%) of the participants in this study had never had a mammogram, whilst one (0.6%) had experienced a mammogram in the past and knew that it should be performed once every two years. This is attributable to the fact that mammography is not available in public hospitals in the region of Vhembe and there are no large-scale awareness campaigns regarding its existence as the majority cannot afford it. Khokhar (2009:249) also indicated, in a study amongst Indian teachers, that mammography is not performed as a routine screening procedure, but is mostly used for those with symptoms or who are at high risk for the disease. Lack of information and knowledge about breast cancer screening practices influences women to miss early detection and treatment opportunities. Screening for early detection and diagnosis of diseases is an important public health principle (Tieng'O *et al*. 2011:3517). Early detection of breast cancer plays the leading role in reducing mortality rates; health education programmes should make women aware of these screening tests, so that they can present themselves early for diagnosis and treatment in order to overcome the burden of the disease. Nurses should emphasise the potential benefits when raising awareness.

Table 2 shows that the participants’ attitudes toward treatment and early detection was positive, however a minority (4.7%) strongly agreed that the treatment worsens the condition and there are still some beliefs that the possibility of a cure is not determined by early detection. Khanjani *et al*. (2012:181) also reported relatively poor knowledge and behaviour amongst female healthcare workers in Tehran. The main reasons given for not performing screening methods were: not feeling a problem; not believing it is necessary; and lack of knowledge. According to the results of the study, being knowledgeable about breast cancer screening methods and when to perform breast cancer diagnostic methods might promote positive health outcomes. Participants who have adequate knowledge regarding how to perform simple life-saving techniques such as BSE, CBE and mammography might be more likely to present at the early stages of breast cancer.

**TABLE 2:** Attitudes of the women toward breast cancer.

Statement	Strongly agree *n* (%)	Agree *n* (%)	Disagree *n* (%)	Strongly disagree *n* (%)
Breast cancer is a curse.	4 (2.7)	37 (24.7)	73 (48.7)	36 (24.0)
Breast cancer is a deadly disease.	75 (50.0)	0 (0.0)	60 (40.0)	15 (10.0)
Breast cancer is a life sentence.	14 (9.3)	72 (48.0)	53 (35.3)	11 (7.3)
Treatment worsen breast cancer.	7 (4.7)	33 (22.0)	75 (50.0)	35 (23.3)
Cure is determined by early detection.	38 (25.3)	70 (46.7)	30 (20.0)	12 (8.0)
Breast cancer leads to depression.	62 (41.3)	79 (52.7)	9 (6.0)	0 (0.0)
If managed well, one can still have a good quality of life.	10 (6.7)	94 (62.7)	45 (30.3)	1 (0.7)
Breast cancer is like any other disease.	20 (13.3)	76 (50.7)	45 (30.0)	9 (6.0)
Women with breast cancer should not be pitied but supported.	38 (25.3)	92 (61.3)	20 (13.3)	0 (0.0)
Clinical Breast Examination should be performed by a female physician.	17 (11.3)	25 (16.7)	74 (49.3)	34 (22.7)

The participants’ attitudes toward treatment and early detection might be influenced by knowledge of breast diagnostic methods and by the participants’ choices on when to seek medical help. Religion, education, occupation and culture might influence the attitudes of the participants regarding who should examine their breast during CBE.

The majority of the women (82.7%) reported that if they noticed any change in their breast, they would consult the medical doctor, whilst 14.0% said that they would consult the traditional doctor and 3.3% would consult the prophet. The issue of religious and cultural belief might play a significant role in the health-seeking behaviour of the participants in this study. McMullin *et al*. (2008:35) reported that native Hawaiians were ‘often offended and resisted participating in research because of the primacy given to the scientific medical model as opposed to lay knowledge and cultural protocols’. This denigration took the form of classifying lay knowledge as myths and misconceptions instead of learning the cultural meaning of cancer in populations. Ethnicity, cultural factors, enabling factors such as having a regular physician to visit, health insurance covering the screening, family and social/family support factors are attributed to health-seeking behaviour. Culture plays a pivotal role in breast cancer screening; amongst black women it can be a barrier as most may not engage in screening programmes because of fear, anxiety and worry as compared to white women, which would prevent them from disclosing the illness or seeking therapy. Social support is important in increasing help-seeking behaviours because having a family or friend can increase the likelihood of being screened.

One hundred and forty-two (94.7%) of the women said that they had never performed breast cancer diagnostic checks before. The results showed no breast cancer screening programme campaigns had ever been conducted in Makwarani Community and participants did not even know how often screening be done. Similar findings were reported by Ojikutu and Adetifa (2009:183), who indicated that the majority (93.3%) of study participants preferred reporting at the hospital or any other health facility within their neighbourhood, whilst only 5.9% preferred to seek the intervention of traditional medical services. This is supported by Mugivhi, Maree and Wright (2009:43), who asserted that most women prefer biomedical treatment. Religious beliefs are particularly dominant amongst black women with a passionate confidence in God, but their mindsets are somehow stuck in attitudes and beliefs such as fatalism, magic, witchcraft and demons. Although Christianity and Islam have replaced traditional religions, the thoughts of the people about life and their attitude to it are still shaped by the old worldview (Akhigbe & Akhigbe 2012:74). Cultural norms and beliefs can act as a barrier to breast cancer treatment. It is therefore important to incorporate culture into interventions designed to increase cancer screening. If we are to develop materials for educational intervention, they should be culturally sensitive as the goal of such a drive would be to increase breast cancer knowledge, decrease cancer fatalism and improve participation in breast cancer screening amongst women.

## Limitations

The research was conducted in one municipality, so the results can therefore not be generalised to others. The results of this research may also not be generalised to larger groups, as they apply only to the rural women of Makwarani who participated in the study.

## Recommendations

There is a need for educational intervention to enhance knowledge about breast cancer, its risk factors and symptoms as well as breast diagnostic examination. The health awareness campaigns should be initiated at primary health level and community home-based carers within communities should be included in these campaigns so as to disseminate information.

Breast cancer information should be accessible to everyone, particularly from the health information centres, through a visible display of posters, information leaflets and health education video clips or recorded health talks whilst clients are awaiting consultations in reception areas.

Additional research is required to change the attitudes of women toward breast cancer and to investigate the various health belief models, as there are still women who believe in the traditional and spiritual dimensions of health and delay seeking help by going to a ‘special doctor’ as they do not understand the concept of speciality in the field of medicine.

Women must be encouraged to practise breast cancer diagnostic examinations regularly. BSE is an economical, simple and basic procedure that can be integrated into all programmes of reproductive health such as adolescent health, pregnancy, post-partum and menopausal women.

## Conclusion

The participants’ level of knowledge about breast cancer was relatively low since the majority (69%) of the participants had never even heard of breast cancer before. This then would make them miss out on the practice of breast cancer screening methods and might also increase their chance of being diagnosed with breast cancer only at the later stages. A negative attitude was found amongst the participants as others still preferred going to traditional and spiritual healers for treatment and did not think that they have personal risk factors.

Poor breast cancer screening was reported in the community since all the participants reported that no screening had ever been conducted in the community, which indicates that dissemination of health information is not working effectively since much information is acquired from the media rather than clinics and/or hospitals. The participants’ practices of breast cancer diagnostic methods were relatively low because only 5.3% had ever practised BSE or CBE or had a mammogram. Education about the importance of early detection in decreasing mortality rates might be of value in raising awareness about the various methods of early detection of breast cancer.
